# Methyl 3-(4-bromo­phen­yl)-2-(1*H*-indol-3-ylmeth­yl)-5-[1-(4-methoxy­phen­yl)-4-oxo-2-phenyl­azetidin-2-yl]-4-nitro­pyrrolidine-2-carboxyl­ate

**DOI:** 10.1107/S1600536808014190

**Published:** 2008-05-17

**Authors:** S. Nirmala, E. Theboral Sugi Kamala, L. Sudha, N. Arumugam, R. Raghunathan

**Affiliations:** a Department of Physics, Easwari Engineering College, Ramapuram, Chennai 600 089, India; b Department of Physics, SRM University, Ramapuram Campus, Chennai 600 089, India; c Department of Organic Chemistry, University of Madras, Guindy Campus, Chennai 600 025, India

## Abstract

In the title compound, C_37_H_33_BrN_4_O_6_, the pyrrolidine ring adopts an envelope conformation. The β-lactam ring is planar and makes dihedral angles of 70.16 (13) and 28.32 (13)° with the phenyl and 4-methoxy­phenyl rings, respectively. The mol­ecular packing is stabilized by intra­molecular C—H⋯O inter­actions and the crystal packing is determined by inter­molecular N—H⋯O hydrogen bonds, and C—H⋯O and C—H⋯π inter­actions.

## Related literature

For related literature, see: Kamala *et al.* (2008 ▶); Lukacs & Ohno (1990 ▶); Sundari Bhaskaran *et al.* (2006 ▶); Suzuki *et al.* (1994 ▶); Yang *et al.* (1987 ▶); Amal Raj *et al.* (2003 ▶); Cremer & Pople (1975 ▶); Nardelli (1995 ▶); Ülkü *et al.* (1997 ▶).
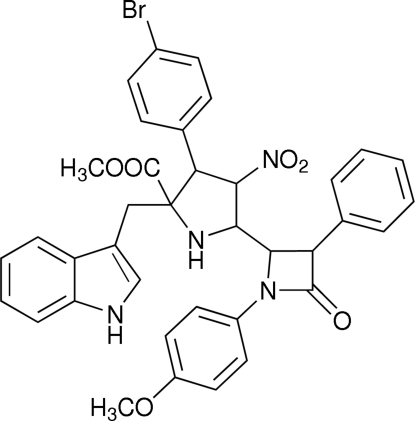


## Experimental

### 

#### Crystal data

C_37_H_33_BrN_4_O_6_
*M* *_r_* = 709.58
Monoclinic, 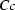

*a* = 11.3988 (4) Å
*b* = 34.8587 (13) Å
*c* = 8.7039 (3) Å
β = 100.982 (2)°
*V* = 3395.1 (2) Å^3^
*Z* = 4
Mo *K*α radiation
μ = 1.26 mm^−1^
*T* = 293 (2) K
0.30 × 0.22 × 0.22 mm

#### Data collection

Bruker Kappa APEX2 diffractometer
Absorption correction: multi-scan (Blessing, 1995 ▶) *T*_min_ = 0.703, *T*_max_ = 0.769
37282 measured reflections
8532 independent reflections
6397 reflections with *I* > 2σ(*I*)
*R*_int_ = 0.028

#### Refinement

*R*[*F* ^2^ > 2σ(*F* ^2^)] = 0.040
*wR*(*F* ^2^) = 0.120
*S* = 1.04
8532 reflections
433 parameters
2 restraints
H-atom parameters constrained
Δρ_max_ = 0.43 e Å^−3^
Δρ_min_ = −0.37 e Å^−3^
Absolute structure: Flack (1983 ▶), 4142 Friedel pairs
Flack parameter: 0.008 (6)

### 

Data collection: *APEX2* (Bruker, 2004 ▶); cell refinement: *APEX2* and *SAINT* (Bruker, 2004 ▶); data reduction: *SAINT* and *XPREP* (Bruker, 2004 ▶); program(s) used to solve structure: *SHELXS97* (Sheldrick, 2008 ▶); program(s) used to refine structure: *SHELXL97* (Sheldrick, 2008 ▶); molecular graphics: *ORTEP-3* (Farrugia, 1997 ▶); software used to prepare material for publication: *PLATON* (Spek, 2003 ▶).

## Supplementary Material

Crystal structure: contains datablocks I, global. DOI: 10.1107/S1600536808014190/rk2086sup1.cif

Structure factors: contains datablocks I. DOI: 10.1107/S1600536808014190/rk2086Isup2.hkl

Additional supplementary materials:  crystallographic information; 3D view; checkCIF report

## Figures and Tables

**Table 1:** Hydrogen-bond geometry (Å, °)

*D*—H⋯*A*	*D*—H	H⋯*A*	*D*⋯*A*	*D*—H⋯*A*
C11—H11⋯O4	0.98	2.27	2.717 (3)	107
C4—H4⋯O4^i^	0.93	2.57	3.186 (3)	124
C31—H31⋯O4^ii^	0.93	2.55	3.399 (3)	152
N1—H1*A*⋯O5^iii^	0.86	2.02	2.820 (3)	155
C18—H18⋯*Cg*^iv^	0.93	2.80	3.641 (4)	151

Symmetry codes: (i) 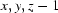; (ii) 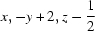; (iii) 
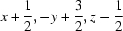
; (iv) 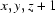. *Cg* is the centroid of the indole benzene ring.

**Fig. 1.**
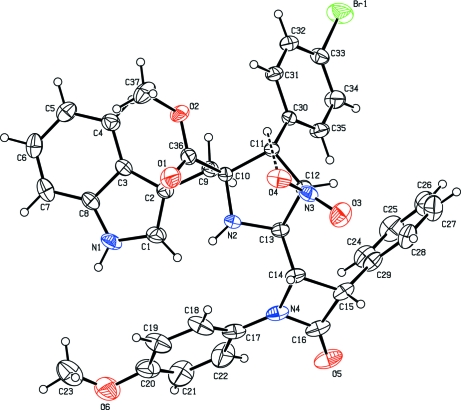
The molecular structure of I. The displacement ellipsoids are drawn at 30% probability level. H atoms are presented as a small spheres of arbitrary radius. The intramolecular H–bond are drawn by dashed line.

**Fig. 2.**
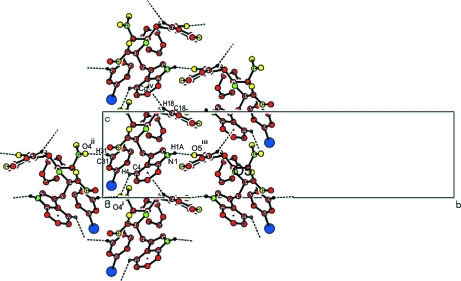
The packing of the molecules viewed down the a axis. Dashed lines indicate hydrogen bonds. H atoms not involed in hydrogen bonds have been ommited for clarity. Symmetry codes: (i) x, y, z - 1; (ii) x, -y + 2, z - 1/2; (iii) x + 1/2, -y + 3/2, z - 1/2; (iv) x, y, z + 1.

## supplementary crystallographic information

### Comment

β–Lactams with a substituent at the N atom, which is easily removable under mild conditions have found wide applicability in the synthesis of bicyclic β–lactam antibiotics (Lukacs & Ohno, 1990). Substituted pyrrolidines have gained much importance because they are the structural elements of many alkaloids. It has been found that they exhibit antifungal activity against various pathogens (Amal Raj *et al.*, 2003). Optically active pyrrolidine derivatives have been used as intermediates in controlled asymmetric synthesis (Suzuki *et al.*, 1994). Since the title compound, (I), also contains an indole unit it may also exhibit some biological activity. In view of these, the structure of title compound is determined to establish the conformation of the molecule (Fig. 1).

The bond lengths and angles in I are agree with those observed in a similar structure (Sundari Bhaskaran *et al.*, 2006; Kamala *et al.*, 2008). The β–lactam ring is planar with its internal angles in the range 84.4 (2) to 95.5 (2)°. The C–C–C bond angle in the β–lactam ring is comparable to the values in related reported structures (Ulku *et al.*, 1997). The bond N4?C16, is shorter than the bond lengths, N4—C14 and N4—C17 and is close to the length of a double bond, a feature observed in β–propiolactam (Yang *et al.*, 1987) and where C and N are *sp*^2^ hybridized.

The methoxy group is coplanar with the C17/C18/C19/C20/C21/C22 benzene ring: dihedral angle C21–C20–O6–C23 = 161.0 (4)°. The methoxyphenyl and the phenyl rings bridged by the β–lactam ring are oriented at an angle of 42.32 (11)° with respect to each other, whereas the β–lactam ring makes a dihedral angles of 28.32 (13)° and 70.16 (13)° with them respectively. The indole moiety is planar and makes a dihedral angle of 38.01 (11)°, 62.8 (7)° and 73.8 (9)° with the β–lactam, bromophenyl and phenyl rings respectively. The nitro–group is orthogonal to indole moiety [89.2 (2)°] and makes a dihedral angle of 66.6 (3)° with the β–lactam ring.

The pyrrolidine ring N2/C10/C11/C12/C13 adopts an envelope conformation, with asymmetry parameters (Nardelli, 1995), Δ*C*_S _(C13) = 0.038 (2) and puckering parameters (Cremer & Pople, 1975) *q_2_* = 0.377 (2)Å and *φ* = 150.8 (3)°. Atom C13 deviates from the mean plane defined by N2/C10/C11/C12 on 0.573 (8) Å.

In the crystal structure of I (Fig.2), adjacent molecules are linked by N—H···O and C—H···O hydrogen bonds into chains. In addition, the packing is stabilized by C—H···*Cg* interactions involving C3/C4/C5/C6/C7/C8 rings with centroid *Cg*.

### Experimental

The β–lactam aldehyde (1 mol) was treated with tryptophanmethylester hydrochloride (1 mol) in the presence of *Et*_3_N (2.5 mol) and anhydrous MgSO_4_ (2 g) in dry dichloromethane (10 ml) at room temperature for 12 h to give the imine. The imine was washed with water and dried over Na_2_SO_4_. The solvent was evaporated under vacuum. The imine (1 mol) was then strirred with silver(I) acetate and *p*–bromo nitrostyrene (1 mol) in the presence of *Et*_3_N (1.2 mol) and molecular sieves in dry toluene (30 ml) at room temperature for 12 h. The reaction mixture was filtered through a plug celite. The solvent was evaporated under reduced pressure and the residue was subjected to column chromatography on silica gel (100–200 mesh), with hexane–ethyl acetate (7:3) as eluent to give the product. The compound was recrystallized from ethyl acetate.

### Refinement

H atoms were placed in idealized positions and allowed to ride on their parent atoms, with C—H = 0.93, 0.98, 0.97 and 0.96 Å for aromatic, methine, methylene and methyl H respectively, and N—H = 0.86 Å, and with U_iso_(H) = 1.5i>U_eq_(C) for methyl and U_iso_(H) = 1.2i>U_eq_(parent C, N) for all other H atoms.

### Crystal data

**Table:**

C_37_H_33_BrN_4_O_6_	*F*_000_ = 1464
*M**_r_* = 709.58	*D*_x_ = 1.388 Mg m^−^^3^
Monoclinic, *C**c*	Mo *K*α radiation λ = 0.71073 Å
Hall symbol: C -2yc	Cell parameters from 13968 reflections
*a* = 11.3988 (4) Å	θ = 2.3–23.4º
*b* = 34.8587 (13) Å	µ = 1.26 mm^−^^1^
*c* = 8.7039 (3) Å	*T* = 293 (2) K
β = 100.982 (2)º	Prism, colourless
*V* = 3395.1 (2) Å^3^	0.30 × 0.22 × 0.22 mm
*Z* = 4	

### Data collection

**Table:**

Bruker Kappa APEX2 diffractometer	8532 independent reflections
Radiation source: fine–focus sealed tube	6397 reflections with *I* > 2σ(*I*)
Monochromator: graphite	*R*_int_ = 0.028
*T* = 293(2) K	θ_max_ = 28.7º
ω and φ scans	θ_min_ = 1.2º
Absorption correction: multi-scan(Blessing, 1995)	*h* = −15→15
*T*_min_ = 0.703, *T*_max_ = 0.769	*k* = −46→46
37282 measured reflections	*l* = −11→11

### Refinement

**Table:**

Refinement on *F*^2^	Hydrogen site location: inferred from neighbouring sites
Least-squares matrix: full	H-atom parameters constrained
*R*[*F*^2^ > 2σ(*F*^2^)] = 0.040	*w* = 1/[σ^2^(*F*_o_^2^) + (0.0682*P*)^2^ + 0.1826*P*] where *P* = (*F*_o_^2^ + 2*F*_c_^2^)/3
*wR*(*F*^2^) = 0.120	(Δ/σ)_max_ = 0.002
*S* = 1.04	Δρ_max_ = 0.43 e Å^−^^3^
8532 reflections	Δρ_min_ = −0.37 e Å^−^^3^
433 parameters	Extinction correction: none
2 restraints	Absolute structure: Flack (1983), 4142 Friedel pairs
Primary atom site location: structure-invariant direct methods	Flack parameter: 0.008 (6)
Secondary atom site location: difference Fourier map	

### Special details

**Table:**

Geometry. All s.u.'s (except the s.u. in the dihedral angle between two l.s. planes) are estimated using the full covariance matrix. The cell s.u.'s are taken into account individually in the estimation of s.u.'s in distances, angles and torsion angles; correlations between s.u.'s in cell parameters are only used when they are defined by crystal symmetry. An approximate (isotropic) treatment of cell s.u.'s is used for estimating s.u.'s involving l.s. planes.
Refinement. Refinement of *F*^2^ against ALL reflections. The weighted *R*–factor *wR* and goodness of fit *S* are based on *F*^2^, conventional *R*–factors *R* are based on *F*, with *F* set to zero for negative *F*^2^. The threshold expression of *F*^2^ > σ(*F*^2^) is used only for calculating *R*–factors(gt) *etc*. and is not relevant to the choice of reflections for refinement. *R*–factors based on *F*^2^ are statistically about twice as large as those based on *F*, and *R*–factors based on ALL data will be even larger.

### Fractional atomic coordinates and isotropic or equivalent isotropic displacement parameters (Å^2^)

**Table:**

	*x*	*y*	*z*	*U*_iso_*/*U*_eq_	
C1	1.0310 (3)	0.82854 (8)	0.0512 (3)	0.0541 (6)	
H1	0.9961	0.8156	0.1244	0.065*	
C2	0.9937 (2)	0.86265 (6)	−0.0140 (3)	0.0406 (5)	
C3	1.07094 (19)	0.87204 (7)	−0.1184 (3)	0.0388 (5)	
C4	1.0793 (2)	0.90284 (8)	−0.2186 (3)	0.0490 (6)	
H4	1.0255	0.9231	−0.2262	0.059*	
C5	1.1678 (3)	0.90276 (10)	−0.3055 (4)	0.0609 (7)	
H5	1.1734	0.9231	−0.3728	0.073*	
C6	1.2498 (3)	0.87257 (11)	−0.2947 (4)	0.0628 (8)	
H6	1.3096	0.8734	−0.3539	0.075*	
C7	1.2437 (2)	0.84207 (9)	−0.1991 (4)	0.0594 (7)	
H7	1.2978	0.8219	−0.1932	0.071*	
C8	1.1543 (2)	0.84186 (7)	−0.1104 (3)	0.0446 (5)	
C9	0.8907 (2)	0.88613 (7)	0.0180 (3)	0.0405 (5)	
H9A	0.8213	0.8697	0.0119	0.049*	
H9B	0.8715	0.9057	−0.0622	0.049*	
C10	0.91742 (18)	0.90557 (6)	0.1798 (2)	0.0352 (5)	
C11	0.81174 (18)	0.93273 (6)	0.2125 (3)	0.0349 (4)	
H11	0.8474	0.9573	0.2510	0.042*	
C12	0.7686 (2)	0.91299 (6)	0.3488 (3)	0.0386 (5)	
H12	0.6811	0.9128	0.3319	0.046*	
C13	0.8177 (2)	0.87176 (6)	0.3464 (3)	0.0385 (5)	
H13	0.7664	0.8584	0.2596	0.046*	
C14	0.8218 (2)	0.84705 (7)	0.4896 (3)	0.0435 (5)	
H14	0.8738	0.8578	0.5822	0.052*	
C15	0.6981 (3)	0.83327 (8)	0.5252 (3)	0.0538 (6)	
H15	0.6945	0.8369	0.6359	0.065*	
C16	0.7435 (3)	0.79315 (9)	0.4961 (4)	0.0637 (8)	
C17	0.9507 (3)	0.78706 (8)	0.4405 (3)	0.0561 (7)	
C18	1.0634 (3)	0.80218 (9)	0.4846 (4)	0.0689 (9)	
H18	1.0722	0.8259	0.5342	0.083*	
C19	1.1637 (4)	0.78329 (10)	0.4579 (5)	0.0771 (10)	
H19	1.2383	0.7949	0.4852	0.093*	
C20	1.1543 (4)	0.74769 (10)	0.3916 (5)	0.0737 (9)	
C21	1.0396 (5)	0.73262 (11)	0.3432 (6)	0.0990 (15)	
H21	1.0312	0.7087	0.2950	0.119*	
C22	0.9395 (4)	0.75174 (10)	0.3643 (6)	0.0893 (13)	
H22	0.8642	0.7413	0.3281	0.107*	
C23	1.3582 (4)	0.74477 (16)	0.3661 (7)	0.1051 (14)	
H23A	1.4164	0.7267	0.3445	0.158*	
H23B	1.3853	0.7559	0.4674	0.158*	
H23C	1.3475	0.7646	0.2881	0.158*	
C24	0.5431 (3)	0.82771 (10)	0.2772 (4)	0.0665 (8)	
H24	0.5881	0.8082	0.2445	0.080*	
C25	0.4380 (3)	0.83898 (13)	0.1818 (4)	0.0798 (10)	
H25	0.4118	0.8263	0.0876	0.096*	
C26	0.3718 (3)	0.86851 (12)	0.2234 (5)	0.0801 (10)	
H26	0.3024	0.8766	0.1571	0.096*	
C27	0.4099 (3)	0.88592 (13)	0.3652 (5)	0.0802 (10)	
H27	0.3645	0.9057	0.3958	0.096*	
C28	0.5143 (3)	0.87489 (11)	0.4641 (4)	0.0685 (8)	
H28	0.5386	0.8874	0.5591	0.082*	
C29	0.5832 (3)	0.84510 (8)	0.4218 (3)	0.0544 (6)	
C30	0.71391 (19)	0.94108 (6)	0.0732 (3)	0.0363 (4)	
C31	0.7291 (2)	0.97173 (7)	−0.0228 (3)	0.0465 (6)	
H31	0.7989	0.9862	−0.0002	0.056*	
C32	0.6435 (2)	0.98119 (8)	−0.1500 (4)	0.0544 (7)	
H32	0.6561	1.0016	−0.2139	0.065*	
C33	0.5397 (2)	0.96064 (8)	−0.1828 (3)	0.0508 (6)	
C34	0.5213 (2)	0.93019 (9)	−0.0920 (4)	0.0586 (7)	
H34	0.4507	0.9161	−0.1153	0.070*	
C35	0.6090 (2)	0.92037 (8)	0.0358 (3)	0.0498 (6)	
H35	0.5969	0.8995	0.0973	0.060*	
C36	1.0328 (2)	0.92886 (7)	0.1965 (3)	0.0419 (5)	
C37	1.1292 (3)	0.97884 (10)	0.0855 (6)	0.0814 (11)	
H37A	1.1137	0.9966	−0.0002	0.122*	
H37B	1.1957	0.9628	0.0749	0.122*	
H37C	1.1476	0.9927	0.1823	0.122*	
N1	1.1279 (2)	0.81582 (6)	−0.0061 (3)	0.0549 (5)	
H1A	1.1659	0.7948	0.0197	0.066*	
N2	0.93345 (17)	0.87641 (5)	0.3021 (2)	0.0397 (4)	
H2	0.9983	0.8644	0.3403	0.048*	
N3	0.8208 (2)	0.93141 (6)	0.5030 (3)	0.0462 (5)	
N4	0.8479 (2)	0.80657 (6)	0.4657 (3)	0.0533 (5)	
O1	1.11802 (17)	0.92443 (7)	0.2958 (3)	0.0690 (6)	
O2	1.02387 (15)	0.95506 (5)	0.0846 (2)	0.0551 (4)	
O3	0.7714 (2)	0.92499 (8)	0.6116 (3)	0.0771 (7)	
O4	0.91172 (19)	0.95038 (6)	0.5130 (2)	0.0617 (5)	
O5	0.7038 (3)	0.76121 (7)	0.4957 (4)	0.0892 (8)	
O6	1.2477 (3)	0.72571 (8)	0.3636 (4)	0.0996 (10)	
Br1	0.42238 (4)	0.974927 (13)	−0.35828 (4)	0.08931 (15)	

### Atomic displacement parameters (Å^2^)

**Table:**

	*U*^11^	*U*^22^	*U*^33^	*U*^12^	*U*^13^	*U*^23^
C1	0.0691 (16)	0.0355 (13)	0.0601 (16)	0.0073 (11)	0.0179 (13)	0.0020 (11)
C2	0.0465 (11)	0.0341 (12)	0.0418 (12)	0.0011 (9)	0.0097 (9)	−0.0032 (10)
C3	0.0393 (10)	0.0357 (12)	0.0389 (12)	0.0034 (9)	0.0016 (9)	−0.0057 (9)
C4	0.0522 (12)	0.0504 (15)	0.0435 (14)	0.0073 (11)	0.0067 (11)	0.0041 (11)
C5	0.0677 (17)	0.0685 (18)	0.0483 (15)	−0.0068 (14)	0.0159 (13)	0.0043 (14)
C6	0.0496 (14)	0.089 (2)	0.0536 (16)	−0.0031 (14)	0.0189 (12)	−0.0149 (16)
C7	0.0468 (13)	0.0708 (19)	0.0589 (17)	0.0136 (12)	0.0055 (12)	−0.0192 (15)
C8	0.0451 (12)	0.0426 (13)	0.0427 (13)	0.0089 (9)	0.0000 (10)	−0.0086 (11)
C9	0.0417 (11)	0.0359 (12)	0.0437 (12)	0.0013 (9)	0.0078 (9)	0.0000 (9)
C10	0.0327 (8)	0.0302 (10)	0.0436 (13)	0.0009 (8)	0.0094 (9)	0.0040 (9)
C11	0.0365 (10)	0.0263 (10)	0.0431 (12)	−0.0011 (8)	0.0103 (8)	0.0000 (9)
C12	0.0389 (10)	0.0354 (11)	0.0419 (12)	−0.0018 (8)	0.0085 (9)	0.0004 (9)
C13	0.0467 (11)	0.0272 (10)	0.0407 (11)	−0.0055 (9)	0.0060 (9)	−0.0010 (9)
C14	0.0566 (13)	0.0337 (12)	0.0378 (11)	−0.0081 (10)	0.0027 (10)	0.0031 (9)
C15	0.0735 (17)	0.0474 (15)	0.0435 (14)	−0.0171 (12)	0.0186 (12)	0.0037 (11)
C16	0.090 (2)	0.0456 (16)	0.0543 (16)	−0.0199 (14)	0.0108 (15)	0.0120 (13)
C17	0.0870 (19)	0.0333 (13)	0.0462 (14)	−0.0035 (12)	0.0080 (13)	0.0045 (11)
C18	0.0743 (19)	0.0456 (16)	0.073 (2)	0.0130 (14)	−0.0217 (15)	−0.0056 (14)
C19	0.079 (2)	0.0572 (19)	0.082 (2)	0.0090 (16)	−0.0163 (17)	0.0014 (17)
C20	0.104 (3)	0.0501 (17)	0.0684 (19)	0.0108 (17)	0.0196 (18)	0.0024 (15)
C21	0.138 (4)	0.0461 (18)	0.128 (4)	−0.017 (2)	0.064 (3)	−0.032 (2)
C22	0.107 (3)	0.0497 (18)	0.122 (3)	−0.0272 (19)	0.050 (3)	−0.026 (2)
C23	0.089 (3)	0.113 (4)	0.105 (3)	0.030 (3)	−0.003 (2)	0.004 (3)
C24	0.0708 (18)	0.0644 (19)	0.0642 (19)	−0.0175 (15)	0.0123 (15)	−0.0084 (15)
C25	0.071 (2)	0.097 (3)	0.065 (2)	−0.0285 (19)	−0.0031 (17)	−0.0118 (18)
C26	0.0577 (17)	0.100 (3)	0.081 (2)	−0.0084 (18)	0.0086 (16)	0.001 (2)
C27	0.0580 (18)	0.105 (3)	0.081 (2)	−0.0011 (17)	0.0217 (17)	−0.003 (2)
C28	0.0642 (18)	0.089 (2)	0.0569 (17)	−0.0115 (16)	0.0242 (14)	−0.0088 (16)
C29	0.0562 (14)	0.0562 (16)	0.0527 (15)	−0.0229 (12)	0.0150 (12)	0.0019 (12)
C30	0.0364 (10)	0.0284 (10)	0.0459 (12)	0.0033 (8)	0.0127 (9)	0.0020 (9)
C31	0.0422 (11)	0.0388 (13)	0.0579 (15)	−0.0039 (9)	0.0082 (11)	0.0151 (11)
C32	0.0532 (14)	0.0470 (15)	0.0642 (17)	0.0012 (11)	0.0142 (13)	0.0201 (13)
C33	0.0498 (13)	0.0488 (14)	0.0513 (14)	0.0102 (11)	0.0038 (11)	0.0088 (12)
C34	0.0437 (12)	0.0555 (17)	0.0703 (18)	−0.0125 (11)	−0.0051 (12)	0.0120 (14)
C35	0.0457 (12)	0.0443 (14)	0.0563 (15)	−0.0075 (10)	0.0017 (11)	0.0168 (12)
C36	0.0369 (10)	0.0364 (12)	0.0532 (13)	0.0003 (8)	0.0110 (10)	−0.0011 (10)
C37	0.068 (2)	0.071 (2)	0.109 (3)	−0.0308 (16)	0.026 (2)	0.015 (2)
N1	0.0716 (14)	0.0342 (11)	0.0589 (14)	0.0197 (10)	0.0124 (11)	0.0003 (10)
N2	0.0430 (9)	0.0298 (10)	0.0463 (11)	0.0066 (7)	0.0086 (8)	0.0070 (8)
N3	0.0578 (12)	0.0345 (11)	0.0452 (11)	0.0106 (9)	0.0070 (9)	−0.0021 (9)
N4	0.0763 (15)	0.0329 (11)	0.0496 (12)	−0.0102 (10)	0.0087 (10)	0.0055 (9)
O1	0.0425 (9)	0.0822 (15)	0.0772 (14)	−0.0107 (9)	−0.0016 (9)	0.0147 (12)
O2	0.0468 (9)	0.0451 (10)	0.0736 (13)	−0.0128 (7)	0.0120 (8)	0.0133 (9)
O3	0.0954 (17)	0.0919 (17)	0.0507 (12)	−0.0014 (13)	0.0306 (12)	−0.0115 (11)
O4	0.0706 (12)	0.0474 (11)	0.0580 (11)	−0.0057 (9)	−0.0105 (9)	−0.0032 (9)
O5	0.115 (2)	0.0477 (12)	0.1045 (19)	−0.0364 (13)	0.0203 (16)	0.0114 (12)
O6	0.128 (3)	0.0694 (16)	0.109 (2)	0.0224 (17)	0.042 (2)	−0.0027 (15)
Br1	0.0742 (2)	0.0972 (3)	0.0835 (2)	0.00646 (19)	−0.01798 (16)	0.0311 (2)

### Geometric parameters (Å, °)

**Table:**

C1—C2	1.351 (4)	C19—C20	1.364 (5)
C1—N1	1.370 (4)	C19—H19	0.9300
C1—H1	0.9300	C20—O6	1.371 (5)
C2—C3	1.419 (3)	C20—C21	1.397 (7)
C2—C9	1.500 (3)	C21—C22	1.364 (6)
C3—C4	1.398 (4)	C21—H21	0.9300
C3—C8	1.410 (3)	C22—H22	0.9300
C4—C5	1.371 (4)	C23—O6	1.420 (6)
C4—H4	0.9300	C23—H23A	0.9600
C5—C6	1.399 (5)	C23—H23B	0.9600
C5—H5	0.9300	C23—H23C	0.9600
C6—C7	1.359 (5)	C24—C25	1.379 (5)
C6—H6	0.9300	C24—C29	1.393 (4)
C7—C8	1.391 (4)	C24—H24	0.9300
C7—H7	0.9300	C25—C26	1.366 (6)
C8—N1	1.357 (4)	C25—H25	0.9300
C9—C10	1.540 (3)	C26—C27	1.369 (6)
C9—H9A	0.9700	C26—H26	0.9300
C9—H9B	0.9700	C27—C28	1.384 (5)
C10—N2	1.458 (3)	C27—H27	0.9300
C10—C36	1.529 (3)	C28—C29	1.393 (5)
C10—C11	1.600 (3)	C28—H28	0.9300
C11—C30	1.511 (3)	C30—C35	1.381 (3)
C11—C12	1.532 (3)	C30—C31	1.388 (3)
C11—H11	0.9800	C31—C32	1.369 (4)
C12—N3	1.505 (3)	C31—H31	0.9300
C12—C13	1.544 (3)	C32—C33	1.366 (4)
C12—H12	0.9800	C32—H32	0.9300
C13—N2	1.453 (3)	C33—C34	1.363 (4)
C13—C14	1.508 (3)	C33—Br1	1.895 (3)
C13—H13	0.9800	C34—C35	1.390 (4)
C14—N4	1.465 (3)	C34—H34	0.9300
C14—C15	1.576 (4)	C35—H35	0.9300
C14—H14	0.9800	C36—O1	1.180 (3)
C15—C29	1.498 (4)	C36—O2	1.325 (3)
C15—C16	1.529 (5)	C37—O2	1.458 (3)
C15—H15	0.9800	C37—H37A	0.9600
C16—O5	1.202 (4)	C37—H37B	0.9600
C16—N4	1.350 (4)	C37—H37C	0.9600
C17—C18	1.374 (5)	N1—H1A	0.8600
C17—C22	1.393 (5)	N2—H2	0.8600
C17—N4	1.408 (4)	N3—O3	1.210 (3)
C18—C19	1.377 (5)	N3—O4	1.218 (3)
C18—H18	0.9300		
			
C2—C1—N1	110.1 (2)	C20—C19—H19	119.8
C2—C1—H1	125.0	C18—C19—H19	119.8
N1—C1—H1	125.0	C19—C20—O6	125.6 (4)
C1—C2—C3	106.7 (2)	C19—C20—C21	117.6 (4)
C1—C2—C9	126.7 (2)	O6—C20—C21	116.8 (3)
C3—C2—C9	126.6 (2)	C22—C21—C20	122.3 (3)
C4—C3—C8	118.6 (2)	C22—C21—H21	118.8
C4—C3—C2	134.5 (2)	C20—C21—H21	118.8
C8—C3—C2	106.9 (2)	C21—C22—C17	119.5 (4)
C5—C4—C3	119.2 (3)	C21—C22—H22	120.3
C5—C4—H4	120.4	C17—C22—H22	120.3
C3—C4—H4	120.4	O6—C23—H23A	109.5
C4—C5—C6	121.1 (3)	O6—C23—H23B	109.5
C4—C5—H5	119.5	H23A—C23—H23B	109.5
C6—C5—H5	119.5	O6—C23—H23C	109.5
C7—C6—C5	121.2 (2)	H23A—C23—H23C	109.5
C7—C6—H6	119.4	H23B—C23—H23C	109.5
C5—C6—H6	119.4	C25—C24—C29	121.2 (3)
C6—C7—C8	118.2 (3)	C25—C24—H24	119.4
C6—C7—H7	120.9	C29—C24—H24	119.4
C8—C7—H7	120.9	C26—C25—C24	121.1 (3)
N1—C8—C7	130.8 (2)	C26—C25—H25	119.5
N1—C8—C3	107.4 (2)	C24—C25—H25	119.5
C7—C8—C3	121.8 (3)	C25—C26—C27	118.4 (4)
C2—C9—C10	112.56 (19)	C25—C26—H26	120.8
C2—C9—H9A	109.1	C27—C26—H26	120.8
C10—C9—H9A	109.1	C26—C27—C28	121.7 (4)
C2—C9—H9B	109.1	C26—C27—H27	119.2
C10—C9—H9B	109.1	C28—C27—H27	119.2
H9A—C9—H9B	107.8	C27—C28—C29	120.3 (3)
N2—C10—C36	108.35 (18)	C27—C28—H28	119.9
N2—C10—C9	109.65 (18)	C29—C28—H28	119.9
C36—C10—C9	109.74 (18)	C24—C29—C28	117.3 (3)
N2—C10—C11	106.06 (16)	C24—C29—C15	121.5 (3)
C36—C10—C11	109.40 (17)	C28—C29—C15	121.2 (3)
C9—C10—C11	113.48 (17)	C35—C30—C31	117.5 (2)
C30—C11—C12	114.28 (18)	C35—C30—C11	124.2 (2)
C30—C11—C10	115.92 (18)	C31—C30—C11	118.3 (2)
C12—C11—C10	103.63 (16)	C32—C31—C30	121.4 (2)
C30—C11—H11	107.5	C32—C31—H31	119.3
C12—C11—H11	107.5	C30—C31—H31	119.3
C10—C11—H11	107.5	C33—C32—C31	119.9 (2)
N3—C12—C11	111.63 (18)	C33—C32—H32	120.0
N3—C12—C13	109.04 (19)	C31—C32—H32	120.0
C11—C12—C13	103.74 (18)	C34—C33—C32	120.7 (3)
N3—C12—H12	110.7	C34—C33—Br1	120.6 (2)
C11—C12—H12	110.7	C32—C33—Br1	118.8 (2)
C13—C12—H12	110.7	C33—C34—C35	119.3 (2)
N2—C13—C14	113.3 (2)	C33—C34—H34	120.4
N2—C13—C12	104.45 (17)	C35—C34—H34	120.4
C14—C13—C12	118.4 (2)	C30—C35—C34	121.3 (2)
N2—C13—H13	106.7	C30—C35—H35	119.4
C14—C13—H13	106.7	C34—C35—H35	119.4
C12—C13—H13	106.7	O1—C36—O2	124.4 (2)
N4—C14—C13	114.1 (2)	O1—C36—C10	125.0 (2)
N4—C14—C15	87.00 (18)	O2—C36—C10	110.6 (2)
C13—C14—C15	116.6 (2)	O2—C37—H37A	109.5
N4—C14—H14	112.3	O2—C37—H37B	109.5
C13—C14—H14	112.3	H37A—C37—H37B	109.5
C15—C14—H14	112.3	O2—C37—H37C	109.5
C29—C15—C16	115.9 (2)	H37A—C37—H37C	109.5
C29—C15—C14	120.7 (2)	H37B—C37—H37C	109.5
C16—C15—C14	84.4 (2)	C8—N1—C1	108.9 (2)
C29—C15—H15	111.1	C8—N1—H1A	125.5
C16—C15—H15	111.1	C1—N1—H1A	125.5
C14—C15—H15	111.1	C13—N2—C10	106.14 (17)
O5—C16—N4	131.8 (4)	C13—N2—H2	126.9
O5—C16—C15	135.1 (3)	C10—N2—H2	126.9
N4—C16—C15	93.1 (2)	O3—N3—O4	124.1 (2)
C18—C17—C22	118.0 (3)	O3—N3—C12	117.0 (2)
C18—C17—N4	122.2 (3)	O4—N3—C12	118.8 (2)
C22—C17—N4	119.8 (3)	C16—N4—C17	130.8 (2)
C17—C18—C19	122.1 (3)	C16—N4—C14	95.5 (2)
C17—C18—H18	119.0	C17—N4—C14	133.2 (2)
C19—C18—H18	119.0	C36—O2—C37	116.1 (3)
C20—C19—C18	120.4 (4)	C20—O6—C23	116.9 (3)
			
N1—C1—C2—C3	0.1 (3)	C24—C25—C26—C27	−2.3 (6)
N1—C1—C2—C9	−179.8 (2)	C25—C26—C27—C28	1.5 (6)
C1—C2—C3—C4	−179.9 (3)	C26—C27—C28—C29	−0.8 (5)
C9—C2—C3—C4	0.0 (4)	C25—C24—C29—C28	−1.5 (4)
C1—C2—C3—C8	0.0 (3)	C25—C24—C29—C15	−179.3 (3)
C9—C2—C3—C8	179.9 (2)	C27—C28—C29—C24	0.7 (4)
C8—C3—C4—C5	0.0 (4)	C27—C28—C29—C15	178.5 (3)
C2—C3—C4—C5	179.9 (3)	C16—C15—C29—C24	−21.8 (3)
C3—C4—C5—C6	−0.4 (4)	C14—C15—C29—C24	77.4 (3)
C4—C5—C6—C7	0.8 (5)	C16—C15—C29—C28	160.5 (3)
C5—C6—C7—C8	−0.8 (4)	C14—C15—C29—C28	−100.3 (3)
C6—C7—C8—N1	−179.4 (3)	C12—C11—C30—C35	−26.5 (3)
C6—C7—C8—C3	0.4 (4)	C10—C11—C30—C35	93.9 (3)
C4—C3—C8—N1	179.9 (2)	C12—C11—C30—C31	152.7 (2)
C2—C3—C8—N1	−0.1 (3)	C10—C11—C30—C31	−86.9 (3)
C4—C3—C8—C7	0.0 (4)	C35—C30—C31—C32	0.0 (4)
C2—C3—C8—C7	−179.9 (2)	C11—C30—C31—C32	−179.3 (2)
C1—C2—C9—C10	72.9 (3)	C30—C31—C32—C33	1.1 (4)
C3—C2—C9—C10	−107.0 (3)	C31—C32—C33—C34	−1.4 (5)
C2—C9—C10—N2	−65.0 (2)	C31—C32—C33—Br1	179.2 (2)
C2—C9—C10—C36	53.9 (2)	C32—C33—C34—C35	0.6 (5)
C2—C9—C10—C11	176.64 (18)	Br1—C33—C34—C35	179.9 (2)
N2—C10—C11—C30	−131.23 (19)	C31—C30—C35—C34	−0.8 (4)
C36—C10—C11—C30	112.1 (2)	C11—C30—C35—C34	178.4 (3)
C9—C10—C11—C30	−10.8 (2)	C33—C34—C35—C30	0.6 (5)
N2—C10—C11—C12	−5.2 (2)	N2—C10—C36—O1	−2.4 (3)
C36—C10—C11—C12	−121.86 (19)	C9—C10—C36—O1	−122.1 (3)
C9—C10—C11—C12	115.22 (19)	C11—C10—C36—O1	112.8 (3)
C30—C11—C12—N3	−133.95 (19)	N2—C10—C36—O2	178.07 (18)
C10—C11—C12—N3	98.99 (19)	C9—C10—C36—O2	58.4 (2)
C30—C11—C12—C13	108.8 (2)	C11—C10—C36—O2	−66.7 (2)
C10—C11—C12—C13	−18.3 (2)	C7—C8—N1—C1	179.9 (3)
N3—C12—C13—N2	−82.7 (2)	C3—C8—N1—C1	0.1 (3)
C11—C12—C13—N2	36.4 (2)	C2—C1—N1—C8	−0.1 (3)
N3—C12—C13—C14	44.5 (3)	C14—C13—N2—C10	−171.01 (19)
C11—C12—C13—C14	163.6 (2)	C12—C13—N2—C10	−40.8 (2)
N2—C13—C14—N4	−68.2 (3)	C36—C10—N2—C13	145.89 (18)
C12—C13—C14—N4	169.0 (2)	C9—C10—N2—C13	−94.4 (2)
N2—C13—C14—C15	−167.4 (2)	C11—C10—N2—C13	28.5 (2)
C12—C13—C14—C15	69.8 (3)	C11—C12—N3—O3	161.8 (2)
N4—C14—C15—C29	−116.5 (3)	C13—C12—N3—O3	−84.1 (3)
C13—C14—C15—C29	−1.0 (4)	C11—C12—N3—O4	−21.0 (3)
N4—C14—C15—C16	0.30 (19)	C13—C12—N3—O4	93.0 (2)
C13—C14—C15—C16	115.8 (2)	O5—C16—N4—C17	−8.2 (6)
C29—C15—C16—O5	−57.5 (5)	C15—C16—N4—C17	173.2 (3)
C14—C15—C16—O5	−178.9 (4)	O5—C16—N4—C14	179.0 (4)
C29—C15—C16—N4	121.1 (2)	C15—C16—N4—C14	0.4 (2)
C14—C15—C16—N4	−0.3 (2)	C18—C17—N4—C16	−147.7 (3)
C22—C17—C18—C19	−1.0 (5)	C22—C17—N4—C16	34.2 (5)
N4—C17—C18—C19	−179.1 (3)	C18—C17—N4—C14	22.5 (5)
C17—C18—C19—C20	−2.8 (6)	C22—C17—N4—C14	−155.6 (3)
C18—C19—C20—O6	−178.0 (4)	C13—C14—N4—C16	−118.3 (2)
C18—C19—C20—C21	4.1 (6)	C15—C14—N4—C16	−0.3 (2)
C19—C20—C21—C22	−1.8 (7)	C13—C14—N4—C17	69.2 (4)
O6—C20—C21—C22	−179.9 (4)	C15—C14—N4—C17	−172.9 (3)
C20—C21—C22—C17	−1.9 (7)	O1—C36—O2—C37	1.6 (4)
C18—C17—C22—C21	3.2 (6)	C10—C36—O2—C37	−178.9 (3)
N4—C17—C22—C21	−178.6 (4)	C19—C20—O6—C23	−17.0 (6)
C29—C24—C25—C26	2.4 (5)	C21—C20—O6—C23	161.0 (4)

### Hydrogen-bond geometry (Å, °)

**Table:**

*D*—H···*A*	*D*—H	H···*A*	*D*···*A*	*D*—H···*A*
C11—H11···O4	0.98	2.27	2.717 (3)	107
C4—H4···O4^i^	0.93	2.57	3.186 (3)	124
C31—H31···O4^ii^	0.93	2.55	3.399 (3)	152
N1—H1A···O5^iii^	0.86	2.02	2.820 (3)	155
C18—H18···Cg^iv^	0.93	2.80	3.641 (4)	151

Symmetry codes: (i) *x*, *y*, *z*−1; (ii) *x*, −*y*+2, *z*−1/2; (iii) *x*+1/2, −*y*+3/2, *z*−1/2; (iv) *x*, *y*, *z*+1.
